# Fluorescence Immunosensor with Phage Antibodies for Heat Shock Protein 70 Detection

**DOI:** 10.3390/bios16040194

**Published:** 2026-03-28

**Authors:** Olga I. Guliy, Sergei A. Eremin, Liliya I. Mukhametova, Evgeniy S. Kozlov, Vyacheslav S. Grinev, Sergey A. Staroverov, Olga A. Karavaeva, Ksenia K. Fursova, Fedor A. Brovko, Lev A. Dykman, Qingyun Liu

**Affiliations:** 1 Institute of Biochemistry and Physiology of Plants and Microorganisms—Subdivision of the Federal State Budgetary Research Institution Saratov Federal Scientific Centre of the Russian Academy of Sciences (IBPPM RAS), Saratov 410049, Russia or grinevvs@sgu.ru (V.S.G.); sastaroverov@gmail.com (S.A.S.); helga1121@yandex.ru (O.A.K.); dykman_l@ibppm.ru (L.A.D.); 2 Department of Chemistry, Lomonosov Moscow State University, Moscow 119991, Russia; saeremin@gmail.com (S.A.E.); liliya106@mail.ru (L.I.M.); 3 Department of Animal Diseases and Veterinary-Sanitary Expertise, Institute of Veterinary Medicine and Pharmacy, Vavilov Saratov State University of Genetics, Biotechnology and Engineering, Saratov 410012, Russia; jkrus19@gmail.com; 4 Institute of Chemistry, Chernyshevsky Saratov National Research State University, 83 Ulitsa Astrakhanskaya, Saratov 410012, Russia; 5 Branch of Shemyakin–Ovchinnikov Institute of Bioorganic Chemistry, Pushchino 142290, Russia; phursova_k@rambler.ru (K.K.F.); brovko@bibch.ru (F.A.B.); 6 College of Chemical and Biological Engineering, Shandong University of Science and Technology, Qingdao 266590, China; skd994185@sdust.edu.cn

**Keywords:** fluorescence immunoassay, circular dichroism, phage antibodies, heat shock proteins, cancer markers, dot immunoassay

## Abstract

The detection of biological markers is critical not only for the early diagnosis of cancer but also for adjustments in antitumor therapy. Rapid, sensitive, and selective detection and monitoring of the content of specific biomarkers in real time are key to point-of-care testing diagnostics. We report the detection of heat shock proteins by fluorescence immunoassay with the appropriate phage antibodies, with a minimum detection limit of 1 ng/mL. The fluorescence immunoassay data were confirmed by dot immunoassay and by circular dichroism studies. The results of the study may help in the adaptation of the fluorescence immunoassay to cancer diagnostics.

## 1. Introduction

Despite the tremendous advances in modern medicine, oncological diseases remain major public health problems worldwide [1]. They pose a serious challenge to all countries and are a primary obstacle to increasing life expectancy. According to the 2022 estimates, about 20.0 million new cancer cases and 9.7 million cancer deaths were recorded worldwide for both sexes [2]. Undoubtedly, such alarming figures draw the attention of the global community to methods of combating cancer pathology.

Currently, the clinical detection of cancer is based primarily on imaging methods or on morphological analysis of presumably disease-affected cells (cytology) or tissues (histopathology). Imaging methods used for cancer detection, including radiography, mammography, computed tomography, magnetic resonance imaging, endoscopy, and ultrasound, are limited in their ability to differentiate between benign and malignant lesions [3]. An important direction in the fight against cancer is the development of early diagnostic methods. It is well known that the earlier the cancer diagnosis, the better the prognosis for its cure [4]. Although a positive result in determining tumor markers does not always indicate the presence of a malignant neoplasm, it may serve as a basis for a more detailed and thorough examination. According to Towards Healthcare Research, the global cancer diagnostics market size is estimated at USD 116.42 billion in 2025 and is expected to reach about USD 199.54 billion by 2034, with a projected compound annual growth rate of 6.17% during the forecast period [5]. In the early diagnosis of cancer, a special place is occupied by methods detecting relevant oncological markers, including heat shock proteins (HSPs).

Human HSPs were initially identified as stress-sensitive proteins necessary for coping with thermal and other proteotoxic stresses. All HSP families encode constitutively expressed genes, such as HSC70 (HSPA8) from the HSP70 family. There are four main HSP families, distinguished by molecular mass: HSP90 (with a molecular mass of about 90 kDa); HSP70 (with a molecular mass of about 70 kDa); small HSPs (with molecular masses ranging from 15 to 30 kDa); and high-molecular-mass HSPs (represented by gp110). All these proteins ensure cell survival under stress, but their functions and tissue specificity differ from group to group, both under normal conditions and under stress [6]. The main characteristics and functions of HSPs have been described elsewhere [7,8].

The functions of HSPs are incredibly diverse and, despite a 30-year history of intense study, are not yet fully understood. HSPs are important in tumor processes, because they participate in cell proliferation, metastasis, and resistance to anticancer drugs [9]. HSP functions are associated with cancer initiation, progression, and metastasis and also with resistance to cancer therapy. Furthermore, HSPs can potentially be used to enhance the effects of chemo-, radio-, and immunotherapy [10,11]. HSPs have numerous clinical applications as biomarkers for cancer diagnosis and prognosis and also as potential therapeutic targets for anticancer treatment [12,13].

HSPs are vital for cancer progression, because they promote tumor cell survival, proliferation, and metastasis. Elevated expression of specific HSPs, such as HSP70 and HSP27, is commonly observed in various malignancies, including lung, breast, and prostate cancer, and it correlates with a poor prognosis and with increased chemotherapy resistance. HSPs stabilize oncogenic proteins, inhibit apoptosis, and modulate the tumor microenvironment, thereby promoting cancer aggressiveness. Recent research highlighted the potential of HSPs as biomarkers for cancer prognosis and treatment response [14]. A growing body of evidence substantiates that HSPs are over expressed in many types of malignant tumors and that their elevated content is enhanced by the hyperactivation of the heat shock factor, which itself contributes to tumor invasion and metastasis [15,16].

The validity of HSPs as tumor-specific biomarkers has been examined in the monitoring of radiation therapy in mouse tumor models and in cancer patients [17,18]. Abe et al. [19] showed that HSP70 is a specific marker for prostate cancer and that its additional determination alongside the prostate-specific antigen (PSA) test is important for identifying patients with early-stage prostate cancer. The authors concluded that PSA screening alone (without HSP70 determination) may result in missed cases of prostate cancer. Other studies have shown that HSP70 is important in the pathogenesis of hepatitis C virus-associated hepatocellular carcinoma [20] and that HSP27 is a potential biomarker for the diagnosis of hepatocellular carcinoma [21].

Analysis of scientific data confirms that the blood serum level of HSP70 may serve as a biomarker for the differential diagnosis of a number of diseases [22]. The threshold values of HSP70 differ greatly, depending on the specific pathology and the detection method [23]. “Normal” HSP70 levels in blood differ greatly from study to study. This is due to differences in the test systems used, patient populations, and preanalytical factors. Depending on the method, levels can range from 0.04 to 49.5 ng/mL. Most solid tumors (lung cancer, pancreatic cancer, glioblastoma) are characterized by a statistically significant increase in serum HSP70 levels. The most dramatic increase is observed in lung cancer (up to 332 ng/mL) [24]. Unlike cancer, cardiovascular diseases show an ambiguous picture. In ischemic heart disease, HSP70 levels may be reduced, as compared with healthy individuals. This may be because intracellular HSP70 fulfills a protective function, and its depletion in blood reflects the severity of the atherosclerotic process. However, in acute conditions, such as pulmonary embolism or cerebrovascular accidents, levels may increase. A study in non-small cell lung cancer showed that HSP70 may serve as a reliable diagnostic marker with a sensitivity of 73% and a specificity of 78% at a cutoff value of 49.48 ng/mL, indicating good discrimination between healthy individuals and those with advanced disease [25]. For example, circulating HSP70 levels can serve as a tumor biomarker for patients with non-small cell lung cancer at advanced stages, according to the Union for International Cancer Control classification [26].

The determination of serum and plasma HSPs by immunoassay methods may be useful for early tumor detection and for cancer treatment monitoring [27]. Therefore, a promising research direction is to develop rapid methods for HSP determination by using immunological (non-invasive) techniques. A key aspect is to choose molecular sensor probes that can be used for specific diagnosis and an appropriate analytic method. Currently, the most sensitive analytic methods are fluorescence assays, and a special position is occupied by fluorescence polarization analysis. It is based on the ability of a fluorescent molecule, after excitation by plane-polarized light, to emit different intensities in different planes. These measurements can be used to measure a fluorescence polarization signal. Equation (1) shows the calculation of polarization (*P*) as the ratio of the difference between parallel ($I_{∥}$) and perpendicular (*I*_⊥_) fluorescence intensities to the total intensity of the emitting light:

(1) $$mP=\;\frac{I_{∥}-I_{⊥}}{I_{∥}+I_{⊥}}\times 1000\;$$

The polarization value *P* is small, and it is more convenient to use millipolarization values (*mP* = *P* × 1000). The observed fluorescence polarization signal depends on the volume of fluorescent molecules at constant temperature and viscosity.

Therefore, fluorescence polarization in a certain range of molecular masses, in which the volume of the molecule is proportional to the degree of fluorescence polarization, can be used to examine biological processes accompanied by a change in the molecular mass of a substance—for example, during protein binding or substrate degradation by the enzymes [28]. The homogeneous fluorescence polarization immunoassay (FPIA) is a one-step assay that does not require secondary antibodies or washing steps. It enables simple and rapid (within minutes) detection of specific antibodies in a test sample. Furthermore, antigens can be detected by competitive FPIA, in which antigen-specific antibodies and a tracer are added to the sample. The minimum detectable analyte amount in a fluorescein-labeled FPIA (about 0.1 ng) is comparable to those yielded by chromatography and ELISA, although it may be limited by sample matrix effects. Owing to its simplicity and rapidity, the FPIA is easily automated and is thus suitable for high-throughput screening in various applications [29]. It is highly sensitive and requires no radioisotopes. The capabilities of the FPIA have been shown by using the fluorescein-labeled ESAT-6 protein for detecting antibodies against *Mycobacterium bovis* in bovine serum [30]; antibodies against *Brucella* [31,32,33]; antibodies against the influenza A virus [34], antibodies against immunoglobulin *E* [35] and proteins [36]; antibiotics such as sulfathiazole [37], sulfonamides, and antibacterial synergists [38]; various toxins [39,40,41,42,43,44,45,46,47,48,49]; sodium benzoate [50]; insecticides [51,52]; atrazine (herbicide) [53]; and biomarkers [54,55,56].

For the detection of an antigen as a bioselective agent (recognition reagent), antibodies or their fragments specific for the target antigen can be used [57,58,59]. In molecular biology, gene-engineering technologies for cloning recognition fragments [hypervariable regions of immunoglobulin G (IgG)] are commonly used. These are relatively inexpensive and can compete with hybridoma technologies in terms of selectivity. One such method is phage display, based on the display of foreign peptides or proteins on the surface of phage particles as part of one of the chimeric coat proteins [60,61].

**Figure 1:**
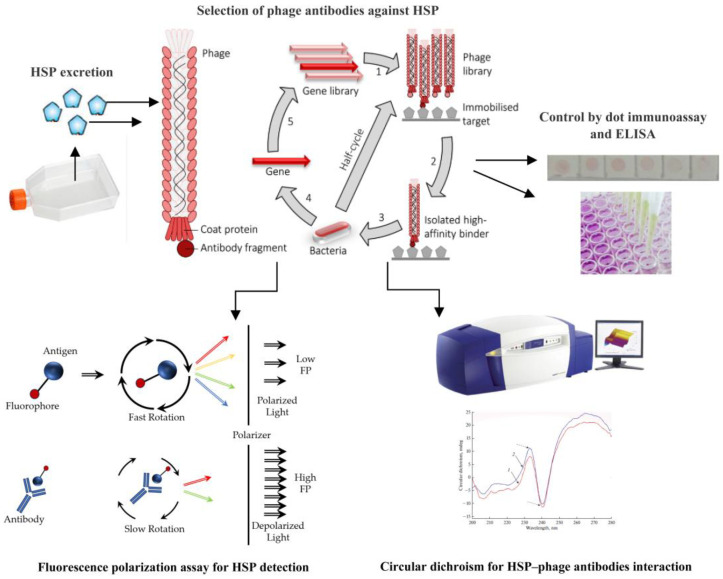
General experimental scheme.

Antibodies obtained by phage display have proven to be highly sensitive components for the detection of antibiotics [62,63,64,65], microbial cells [66,67,68,69,70], viruses [71,72,73,74,75], heavy metal toxicological targets [75], and diagnostically important antigens, including tumor markers [76,77,78,79]. An undoubted advantage of phage antibodies is their excellent reproducibility; i.e., each subsequent antibody obtained for the detection of HSPs will be identical to the previous ones. This is critically important for a stable diagnostic test system [80]. Phage antibodies have a low molecular weight, and their fluorescently labeled derivatives (tracers) can be used as recognizing reagents in the FPIA. The detection of cancer-associated biomarkers by using the FPIA and phage antibodies as recognition reagents specific to the target marker is a new research direction aimed at expanding the application of fluorescence polarization analysis for early cancer diagnosis. We developed a rapid method for determining cancer biomarkers, with HSPs as an example, by using the fluorescence immunoassay with biosensor tracers and phage antibodies specific for the target antigen. Figure 1 shows the general experimental scheme.

## 2. Materials and Methods

### 2.1. Cell Culture Conditions and Obtainment of a Thermostable Antigen Fraction

HSPs were isolated from neoplastic cells of spontaneously diseased cats admitted with a confirmed diagnosis of mammary adenocarcinoma to Vavilov Agrarian University’s veterinary clinic (Saratov, Russia). Animal-related experiments were conducted in accordance with the NIH Guidelines for the care and use of laboratory animals [81]. A detailed method for HSP70 isolating is described in [82].

To conduct a cross-reactivity test with the homologous protein HSP70, we used HSP70 isolated from Chinese hamster ovary (CHO) cells. The cells were cultured in Dulbecco’s modified Eagle’s medium (DMEM; BioLot, Saint Petersburg, Russia) supplemented with 10% fetal bovine serum (BioLot, Russia), penicillin (100 units/mL), streptomycin (100 μg/mL) and L-glutamine (292 μg/mL).

The permit document for the conduct of animal studies was Charter No. 66-y, approved by order of the Ministry of Agriculture of the Russian Federation on 18 June 2015. Care, maintenance, and handling of animals were in accordance with the Guide for the Care and Use of Laboratory Animals of the Ministry of Health of the Russian Federation, the European Convention for the protection of vertebrate animals used for experimental and other scientific purposes, and the legislation of the Russian Federation. The diagnosis was made on the basis of clinical signs and an analysis of cytopuncture material studies. The final diagnosis was made on the basis of the histopathological picture of surgical specimens, as described in [79].

### 2.2. Preparation of HSP-Specific Phage Antibodies

These were prepared by using a non-immune phage library displaying human single-chain variable fragments (scFv) (a diversity of 10^9^ independent clones) [83]. Selection was done as follows: the antigens isolated from tumor cells were immobilized on a polyvinylidene fluoride membrane (Millipore, Burlington, MA, USA) overnight at 4 °C. The membrane was blocked with 2% dry milk in phosphate-buffered saline (PBS) at room temperature for 1 h on a shaker, washed three times in PBS, and used for antibody selection. The membrane with the immobilized antigen was incubated with phage particles (200 μL) at room temperature for 1 h on a shaker. Non-specifically adsorbed phage particles were washed four times in PBS. The remaining phage were eluted with 0.1 M glycine–HCl buffer (pH 2.5; 500 μL) at room temperature on a shaker. The phage eluate was collected and neutralized with 500 μL of 1 M Tris–HCl (pH 9.5).

*Escherichia coli* TG1 (IBPPM RAS Collection of Rhizosphere Microorganisms, http://collection.ibppm.ru) was grown to the logarithmic phase at 37 °C without shaking to form F pili. The neutralized eluate (1 mL) was used to infect *E. coli* TG1. After 30 min of incubation at 37 °C without shaking, the infected cells were pelleted by centrifugation at 3500× *g* for 10 min and were subcultured onto 2xYT medium (Sigma-Aldrich, St. Louis, MO, USA) with 100 μg/mL ampicillin and 1% glucose. The culture was incubated on a shaker at 37 °C overnight. One mL of the culture was subcultured in 10 mL of fresh 2xYT medium with 100 μg/mL of ampicillin and 1% glucose, and the mixture was incubated on a shaker at 37 °C until the logarithmic growth phase was reached. The cells were then incubated at 37 °C for 30 min without shaking, and M13KO7 helper phage particles were added. M13KO7 (family Inoviridae, commercial agent produced by Stratagene, Solna, Sweden), constructed from wild-type phage M13, were used as a model system [84,85]. The infected cells were centrifuged at 3500× *g* for 10 min after being incubated at 37 °C for 30 min without shaking. They were then subcultured in 2xYT medium containing 100 g/mL of ampicillin, 50 g/mL of kanamycin, and 100 g/mL of iso-propyl-D-1-thiogalactopyranoside. The culture was shaken at 30 °C for the entire night. Centrifugation at 3500× *g* for 20 min was used to pellet the cells, and centrifugation at 10,000× *g* for 20 min was used to further clarify the liquid supernatant. After adding 20% of the volume of a PEG/NaCl solution to the liquid supernatant, the combination was incubated at 0 °C until a precipitate became apparent. Centrifugation at 20,000× *g* for 15 min was then used to pellet the precipitate. The pellet was dialyzed against PBS via a dialysis membrane at 4 °C for 24 h after being resuspended in 1 mL of PBS. There were three rounds of selection in a row. A detailed description of the preparation procedure can be found elsewhere [78].

The phage particle concentration was measured spectrophotometrically:

(2) $$C=\frac{\left(A_{269}-A_{320}\right)\times 10^{14}}{15}$$

where *A*_320_ and *A*_269_ are the absorbance values for phage particle suspensions at 320 and 269 nm.

The measurements were made at the Simbioz Center for the Collective Use of Research Equipment (IBPPM RAS, Saratov, Russia).

### 2.3. FITC Labeling of Phage Antibodies

The phage antibodies (Rec-Ab) that resulted from dialysis were labeled with fluorescein isothiocyanate (FITC) as follows: before labeling, the antibodies were dialyzed against carbonate–bicarbonate (17.3 g sodium hydrogen carbonate and 8.6 g sodium carbonate in 1000 mL of water) at 4 °C overnight. Then, 1 mg of FITC was dissolved in 2 mL of 0.1 M PBS (pH 9)and FITC was added drop wise to the phage solution with constant stirring. Conjugation was continued in the dark at 4 °C for 18h. After the process had ended, excess dye was removed by filtration on a Sephadex G-25 column and the solution containing labeled phage was applied to the column and was eluted in 10 mM PBS (pH 7.4) containing 0.14 M NaCl. Finally, the FITC-labeled phage antibodies were concentrated by ultrafiltration by using Amicon Ultra filters (pore size, 3 kDa) (Merck KGaA, Darmstadt, Germany), frozen, and stored at −20 °C before use. The phage antibodies and the FITC-labeled phage antibodies were characterized spectrophotometrically on a Biochrom WPA Lightwave II spectrophotometer (Biochrom Limited, Cambridge, UK).

The (RecAb)/FITC ratio was determined from the absorption spectra for the labeled antibodies in 10 mM PBS (pH 7.4), by Equation (3):

(3) $$\frac{RecAb}{FITC}=\;\frac{A_{492}\times \varepsilon_{RecAb}}{{(A}_{280}-C\times A_{492})\times \varepsilon_{FITC}}$$

where $A_{492}$ is the absorbance of the conjugate RecAb-FITC at the wavelength of maximum FITC absorption (492 nm), $A_{280}$ is the absorbance of the sample at the wavelength of maximum antibody absorption (280 nm), $\varepsilon_{HSP}$ is themolar extinction coefficient of the RecAb at 280 nm (40,170 M^−1^ × cm^−1^, this value is calculated based on the amino acid composition (number of tryptophan, tyrosine, and cysteine residues) using tools like ProtParam (Swiss Institute of Bioinformatics, Lausanne, Switzerland) [86], $\varepsilon_{FITC}$ is the molar extinction coefficient of FITC at the wavelength of maximum absorption (72,000 M^−1^ × cm^−1^), and *C* is the correction factor for FITC at 280 nm (*C* = 0.35). The approximate HSP/FITC ratio was 4:1.

### 2.4. Fluorescence Polarization Immunoassay with Phage Antibodies

Initially, solutions of FITC-labeled phage antibodies were prepared in 10 mM PBS (pH 7.4) containing 150 mM NaCl, so that the fluorescence intensity of the solutions would not exceed 200,000 units. The phage antibody concentration was 2.5 nM. One mL of the working solution of fluorescently labeled nanobodies was placed in a borosilicate glass test tube (10 × 75 mm), and 10 μL of different concentrations of the antigen solution (0–60 μg/mL) was added. After 5 min of incubation at room temperature, the change in the fluorescence polarization signal (*mP*) and the intensity of the test solution were measured. Measurements were made on a Sentry-200 portable fluorimeter (Ellie LLC, Germantown, WI, USA; λ_ex_/λ_em_, 495/530 nm), as described previously [35].

### 2.5. Dot Immunoassay

The ability of the selected phage to bind to the HSPs was examined by dot immunoassay and by a secondary labeling strategy [13].

### 2.6. ELISA

This was done as described elsewhere [13]. The reaction was stopped with 100 µL of 0.1 M sulfuric acid, and the results were recorded on a Multiskan Ascent+ microplate spectrophotometer (Thermo Scientific, Waltham, MA, USA) at 490 nm.

Goat anti-rabbit IgG conjugated to horseradish peroxidase (Sigma-Aldrich, USA) and *o*-phenylenediamine (Fluka, Buchs, Switzerland) were used.

### 2.7. Circular Dichroism (CD) Spectra Measurements

These were made on a Chirascan spectrometer (a circular dichroism spectrometer, which uses polarized light; Applied Photophysics, Leatherhead, UK) equipped with a continuous purge system with high-purity nitrogen (99.996%; oxygen volume fraction, no more than 0.001%). A 101-QS P quartz cuvette (Hellma Analytics, Müllheim, Germany), designed for polarization measurements and free from internal stress, was used to record the spectra (optical path length, 1 mm). The measurement range was 180–280 nm, with a 1nm step, and the scanning speed was 4 nm s^−1^. The measured ellipticity is presented in millidegrees (mdeg). For comparison, the spectrum of the corresponding solvent was prerecorded. The measurements for all solutions were made in a quartz cell under similar conditions. All measurements were made at room temperature at the Simbioz Center for the Collective Use of Research Equipment (IBPPM RAS, Saratov).

### 2.8. Statistics

Data were processed with SigmaPlot 11 software (Systat Software, Inc., San Jose, CA, USA).

## 3. Results

One targeted research effort to develop early cancer diagnostic methods is to make molecular probes that can specifically bind to biological markers. Previously, we developed and characterized a technology for preparing phage antibodies (RecAb–FITC) specific for HSPs isolated from spontaneously infected animals with a confirmed cancer diagnosis. Because it had previously been shown that the specificity and sensitivity of phage antibodies increase after round four of antibody selection, we conducted here five rounds of antibody selection. The titer of the phage antibodies was 1:2560.

**Figure 2:**
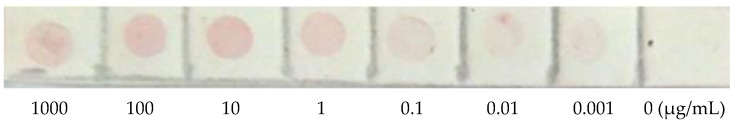
Dot immunoassay of HSP70 by using phage antibodies to the HSP70 from the tumor of a spontaneously infected animal. The antibodies were obtained after five selection rounds.

Subsequently, by dot immunoassay, we monitored the interaction of the RecAb–FITC phage antibodies with the HSPs. Gold nanoparticles complexed with antiphage antibodies bound to the HSP (antigen)-specific anti-HSP phage antibody complex. This could be visually observed as a series of pink spots (Figure 2). The minimum visually detectable antigen concentration, as found by dot immunoassay with the prepared phage antibodies, was 0.001 µg/mL (1 ng/mL).

**Figure 3:**
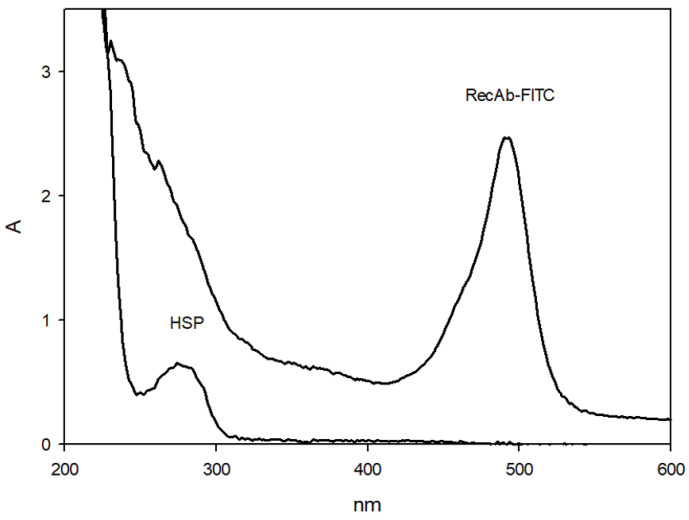
UV–vis spectra for RecAb–FITC and Ag-HSP.

We next examined the use of the FIA to detect the interaction of phage antibodies with Ag-HSPs. A fluorescently labeled derivative of phage antibodies, RecAb–FITC, was obtained; Figure 3 shows its UV–vis spectrum.

**Figure 4:**
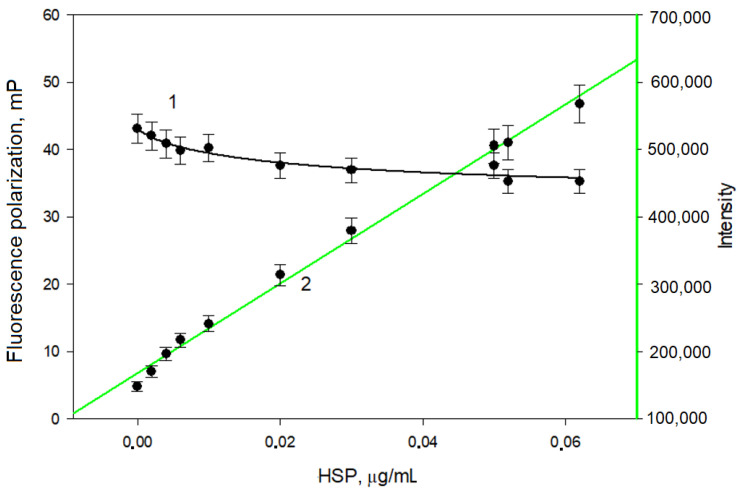
Changes in the polarization (*mP*, curve 1) and fluorescence intensity (Intensity, curve 2) values after binding of RecAb–FITC to Ag-HPS. Temperature, 25 °C; pH 7.4.

As indicated by the absorption peak at 495 nm, the phage antibodies were successfully conjugated to fluorescein. Subsequently, a solution of RecAb–FITC was prepared (concentration, 2.5 nM), and changes in the polarization signal (*mP*) and in the fluorescence intensity (Intensity) were monitored as the Ag-HSP concentration was increased. Figure 4 shows the results.

As seen from Figure 4, when the Ag-HPS concentration increased during the interaction with the phage antibodies, the fluorescence polarization signal changed slightly. Apparently, the interaction of RecAb–FITC with Ag-HPS led to no significant increase in the molecular weight of the formed complex, which is why no change in the fluorescence polarization signal was observed. However, with increasing Ag-HPS concentration, the fluorescence intensity of the solutions increased, which indicates an interaction between the antibodies and the antigen.

The observed fluorescence increase upon antigen–antibody binding may be due to a combination of factors:

(i) A change in the local polarity/hydrophobicity around the fluorophore when the antibody binds to the antigen. As a result, the quantum yield [87,88] and the polarity of the microenvironment [89] may change.
(ii) The displacement of the fluorophore from aqueous quenchers (e.g., dissolved oxygen or buffer components) upon complex formation [88,90,91] and photoinduced electron transfer [92].
(iii) Possible restriction of intramolecular rotations (if the fluorophore is environmentally sensitive; e.g., molecular rotor behavior) [93] and violation of intramolecular dimer formation [94].

The enhancement of fluorescence upon antigen–antibody binding is a well-documented phenomenon that can be attributed to several interrelated factors. Therefore, while our study focuses on analytical application, the proposed mechanism is firmly grounded in these established photophysical principles.

The observed lack of significant changes in polarization may be due to compensation effects: increasing the mass of the complex (antibody + antigen) reduces polarization, but the release of the fluorophore and its increased local mobility increases polarization. These effects may cancel each other out. The instrument settings used in this study may not have corresponded to the fluorophore rotation kinetics under our conditions.

The fluorescence of fluorescein depends greatly on its immediate environment. It is possible that in an antibody–fluorescein conjugate, the fluorophore can move and rotate relatively freely, thereby coming into close proximity with intrinsic antibody “quenchers,” such as tryptophan residues, histidine residues, and disulfide bonds. Upon close contact, the excitation energy of fluorescein can be transferred to these groups by way of non-radiative pathways, which quenches its fluorescence. Thus, in the native (free) antibody, fluorescein may be in an unfavorable environment, and its fluorescence is quenched. However, the binding of the antibody to its specific antigen may lead to a change in the microenvironment of fluorescein. It may move away from the amino acid residues that quench its fluorescence and may find itself in a more hydrophobic and rigid environment. As a result, fluorescence is restored (dequenched).

Fluorescence polarization is directly proportional to the rotational relaxation time of the molecule, which, in turn, depends on the hydrodynamic volume of the particle. When a phage–antibody complex is formed (which is essentially a bacteriophage presenting an antibody fragment), the object greatly exceeds both free fluorophore and classical IgG in size. High background in FIA analysis usually occurs because the free (unbound) ligand itself is polarized abnormally highly (e.g., owing to aggregation, adhesion to the walls, or solution viscosity). Because a phage is a huge particle (as compared with the dye molecule), the rotational mobility of the free labeled component (be it antigen or phage) remains high and the polarization is low. The formation of a phage-involving complex makes rotation barely possible, which results in a maximum signal increase. Using such a large label extends the dynamic range, as compared with assays based on individual IgG antibodies. In a classic immunoassay, the antibody–antigen complex is only 2–3 times larger than the antibody, and the polarization difference may be small. Here, however, the difference is maximal for a given fluorophore. Therefore, the large size of the phage–antibody complex in the context of fluorescence polarization is not a hindrance but an advantage. It allows one to achieve a maximum dynamic range and preserve a low background signal, making this method highly sensitive for detecting interactions. Even if the background polarization of free phage is higher than that of free fluorescein, a statistically significant signal increase is still obtained upon binding. The key is to control the concentration to avoid viscosity effects.

From the obtained data, a calibration graph was constructed to determine Ag-HPS (Figure 4). The detection range for the antigen was 0.002–0.05 µg/mL, and the detection limit was 0.001 µg/mL (1 ng/mL).

Thus, after the interaction of RecAb–FITC with the HSPs at a constant *mP* value, an increase in the fluorescence intensity is observed that is proportional to the antigen concentration. Consequently, the HSP antigen can be determined on the basis of the increase in fluorescence by using RecAb–FITC. The minimum detectable HSP concentration was 0.001 µg/mL. Thus, we developed an FIA for HPS determination on the basis of the FPIA.

Comparison of the detection limit with those afforded by conventional methods shows that the HSP70detection limit yielded by ELISA ranges from 0.2 to 125 ng/mL, depending on the kit. Specifically, the widely used Thermo Fisher HSPA4 kit has a detection limit of 2 ng/mL, whereas the specialized kit for detecting HSP70 in milk has a detection limit of 125 ng/mL. Western blotting, being a semi-quantitative method, does not afford such accuracy in high-throughput analysis. The sensitivity of ELISA to HSPs differs greatly from that of Western blotting, with ELISA typically being one to three orders of magnitude more sensitive. Furthermore, Western blotting requires higher initial protein concentrations to obtain a clear signal [95].

Therefore, the main advantage of the FIA is the combination of a low detection limit with rapidity and quantitative accuracy, which distinguishes it from traditional approaches.

We used FIA and ELISA to test cross-reactivity with the homologous protein HSP70 from CHO cells. No significant signal was observed down to 1 μg/mL, which eliminated the possibility of false-positive results owing to cross-reactivity.

To confirm the FIA results for the interaction between phage antibodies and Ag-HPS, we used the circular dichroism (CD) method. CD is a specific case of elliptical dichroism, in which right-handed and left-handed circularly polarized light is absorbed in different proportions by the substance [96]. An advantage of CD spectroscopy is that it can detect specific binding at low concentrations of the components being analyzed in a biological sample (up to 20 µg/mL) [97].

**Figure 5:**
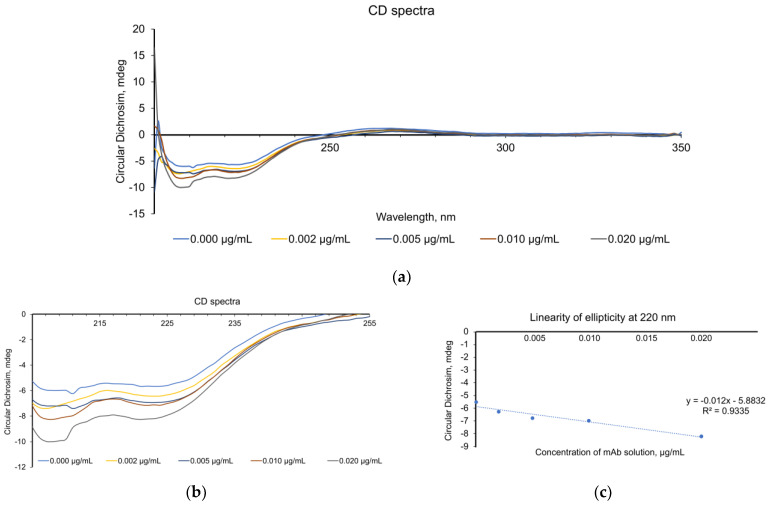
(**a**) CD spectra (all wavelengths). (**b**) At 0–255 nm. (**c**) Linearity of the decrement at 220 nm.

The presented CD spectra show significant conformational changes in the phage antibody molecules after their interaction with the HSPs. The initial spectrum of the phage antibodies (0.002 µg/mL) had a profile typical of *β*-rich proteins, with two minima at ~220 and ~205 nm [Figure 5a]. Then, with the addition of increasing HSP70 concentrations, the spectrum changed. Figure 5a shows the data obtained up to a wavelength of 350 nm. The changes in the near-UV region (250–300 nm) were minimal, which indicates preservation of the overall tertiary structure of the complexes despite the changes in the local environment of the aromatic amino acid residues. For clarity, Figure 5b shows the data up to 250 nm. Figure 5c shows the signal changes at 220 nm. It can be seen that linearity was maintained within the antigen concentration range of 0–0.020 μg/mL, with an *R*^2^ correlation coefficient of 0.9335. The CD spectra allowed us to detect fine conformational changes during the protein–protein interaction in solution.

After sequential addition of an HSP solution, the profile of the CD spectra changed. The addition of 1–10 µL of the HSP solution led to minor spectral changes, which indicates the initial stage of the antigen–antibody interaction. The addition of the first HSP portions resulted in a decrease in the intensity of the negative peak at ~220 nm, which may indicate partial destabilization of the *β* structures within the phage antibodies. A further increase in the antigen concentration (0.002–0.050 µg/mL) produced greater spectral changes, indicating the formation of stable complexes. The greatest changes were observed in the 200–230nm region, which indicates rearrangement of the secondary structure of the protein molecules. On a further increase in the HSP concentration, the spectral profile got stabilized and a new equilibrium state was formed. The observed changes in the CD spectra indicate the formation of specific complexes between phage antibodies and HSPs through a conformational selection mechanism. The gradual nature of the changes suggests a multistage binding process with intermediate conformational states, which is typical of antibody–antigen interactions.

The decreased CD intensity signal in the 215–225nm region may have reflected induced conformational changes in the complementary-determining regions (CDRs) of phage antibodies upon recognition of the HSP epitopes. The obtained results are consistent with the modern understanding of the mechanisms of the antibody–antigen interaction, as studied by CD spectroscopy. The conformational changes during the formation of a protein–protein complex may be evaluated by changes in the far-UV region of the spectrum [98]. Similar changes in the 220nm region were observed in studies of interactions between various scFv fragments and antigens [99].

The nature of the observed changes corresponds to the data indicating that antigen recognition occurs through a conformational adaptation mechanism with mutual structural rearrangements of both the antibody and the antigen [99].

Similar changes in CD spectra during antibody interactions with the HSP27 protein, including changes in *β*-structure content, were described earlier [100]. The phage antibodies used in this work were scFv that retained the antigen-binding ability of full-length antibodies while having a much lower molecular mass (~27 kDa) [101]. This ensured higher sensitivity of the CD method to conformational changes, as compared with the use of full-length immunoglobulins.

The obtained CD spectroscopy data showed successful formation of specific complexes between HSP-specific phage antibodies and their target antigens. The observed conformational changes indicate a functional interaction, with the structural integrity of the protein components being preserved. This confirms not only the potential of these phage antibodies for practical application but also the FIA data.

Thus, we have shown for the first time that the FIA with phage antibodies can be used for the detection and determination of HSPs. One important aspect is to choose an optimal antibody–tracer pair [45], and this was achieved by testing various antibody–tracer combinations in terms of uniform cross-reactivity and sensitivity. The FIA results were confirmed by dot immunoassay and by CD measurements. One can conclude that the antigen-specific phage antibodies interact with the corresponding HSP-binding sites and can be used as recognition components for HSP determination.

## 4. Discussion

As pointed out previously, early diagnosis of oncological diseases is a promising research direction, in which rapid cancer detection methods play an important part. Tumor markers can provide a wide range of information crucial for cancer treatment, namely the type of cancer, the stage of the disease, and the assessment of the prognosis for pathology development and treatment.

An elevated content of a tumor marker does not mean a patient has cancer. Non-cancerous conditions can sometimes cause an increase in tumor marker content. Moreover, not all patients with a specific type of cancer will have an elevated content of the tumor marker associated with that cancer type. Therefore, for cancer diagnosis, tumor marker measurements are commonly combined with other tests, such as a biopsy or imaging. Tumor markers that indicate whether a patient is a candidate for a specific targeted therapy are sometimes called cancer treatment biomarkers. Biomarkers are usually measured in tumor tissue samples. However, tumors can shed cells or fragments of biological material into blood, and this can be evaluated by a test called liquid biopsy.

Periodic (or “serial”) measurements of a marker during treatment can indicate the tumor’s response to therapy. Periodic measurement of tumor markers after treatment completion can be used to check for the likelihood of recurrence [102].

Immunological detection methods are crucial for identifying specific biomarkers for the diagnosis, prognosis, and treatment of diseases in precision medicine. With the recent shift in focus toward personalized treatment, immunological methods based on the antigen–antibody interaction provide a unique platform for obtaining accurate and reliable information about the presence of specific disease markers [103].

In the development of methods for detecting cancer markers, immunological methods, including the FPIA, have attracted particular attention. Owing to its fundamental signal readout principle, the fluorescence polarization system offers several advantages over fluorescence-based methods. The FPIA is a homogeneous method, and it does not require separation of the bound fraction from the free fraction. It is easily automated and, thanks to portable equipment, can be used in rapid on-site tests. Furthermore, only one reagent (a fluorescently labeled conjugate) is required to detect high-molecular-weight antigens. By using the FPIA, a new recognition reagent (fluorescently labeled phage antibodies) was obtained against HSPs, and an FIA was developed for detecting HSPs. The main advantage of this FIA is that it is rapid and sensitive, does not require lengthy manipulations, and can be applied anywhere. Furthermore, it is inherently quantitative and permits an objective assessment of disease severity. Unlike heterogeneous assays requiring separation stages to obtain a target-dependent signal, homogeneous assays enable the detection of target molecules without laborious separation steps through the use of specialized reporters and can be applied in biosensing (immunosensing) for the rapid and sensitive detection of low-molecular-mass antigens [104].

The developed FIA is highly sensitive, specific, and practical, because there is no need to use expensive and hazardous reagents (e.g., radioactive materials). These advantages contribute to its adaptation to the biomedical field to determine drugs, hormones, proteins, and antigens and also to identify antibodies [105,106]. The development of various fluorescent probes and tools contributes to the constant advancement of the FIA. Recent accomplishments and challenges in the development of methods, recognition elements, and fluorophores, in multi target analysis, and in their application to actual samples have been shown previously [107,108]. To date, numerous FIA-related technologies have been developed with high detection sensitivity and various measurable properties, including fluorescence resonance energy transfer immunoassay, FPIA, and time-resolved fluorescence immunoassay [54].

As seen from the presented data, most research has been focused on the determination of antibodies [109], the use of aptamers [110,111], the use of the noncompetitive fluorescence polarization immunoassay, and the use of peptides as tracers [53].

An additional advantage of the method is that it can be used to determine low-molecular-mass substances, e.g., low-molecular-mass mycotoxins (less than 1 kDa), including aflatoxins, fumonisins, group B trichothecenes (primarily deoxynivalenol), ochratoxin A, and zearalenone [112]. For oncology, the capabilities of the FIA were presented on the basis of a multicolored quantum dot nanosensor for the multiplex detection of two tumor markers in a homogeneous format [54]. Research showing how the FIA can be combined with phage antibodies to detect HSPs is absent in the literature and is shown in this work.

The results obtained in this study may be useful for the development of a diagnostic system for cancer diagnostics in humans. The justification for extrapolating data from the feline model to humans is based on comparisons between genomics and oncology. The evolutionary conservatism of HSP70 chaperones, determined in this work, is highly conserved molecules. Studying their structure and function in spontaneous feline tumors allows us to identify universal mechanisms of oncogenesis, which are independent of species. The presented results are based on the One Health paradigm, in which feline mammary cancer is considered not simply a model but an analog of the human disease [113]. Feline mammary cancer is recognized by the international scientific community as one of the most relevant models for studying human breast cancer [114]. Unlike transplantation or induced mouse models, feline tumors develop in the context of a natural immune system, in an environment with normal microclonal diversity, and under the same environmental and age-related factors as in humans. Feline mammary adenocarcinoma shows histopathology that is similar to that in humans, a high rate of metastasis, and similar mechanisms of tumor progression. This makes the feline spontaneous cancer model, used in this study, more representatives for studying biomarkers associated with tumor aggressiveness than classical mouse models [115]. The results obtained by using feline samples represent a necessary fundamental step in focusing biomarker research in humans.

Further, recombinant phage antibodies, as used for HSP70 detection in combination with the FIA, ensure the critically important stability and reproducibility of results. This is essential for both scientific research and further adaptation of methods to diagnostic needs. The use of scalable bacterial systems for antibody preparation greatly reduces the cost of test systems. For example, the yield of functional Fab fragments against HSP70 was increased more than 100-fold through co-expression of chaperones, which enabled the preparation of more than 15 mg of pure protein per liter of culture in a laboratory fermenter [80].

The availability of portable devices could extend diagnostics beyond the laboratory, making this method accessible at the point of need. Further research will focus on adapting the method to detect cancer markers in complex biological matrices (e.g., lymph, blood serum) and on platform miniaturization (e.g., smartphone-based detection).

Blood serum is a complex matrix containing proteins (albumins, globulins), lipids, and other components that can potentially influence fluorescence polarization through autofluorescence, protein binding, polarized light scattering, fluorescence quenching, and other factors. In our experiments with simulated serum, we specifically evaluated the extent of these effects. By optimizing the dilution ratio (a coefficient was chosenthat minimizes matrix influence while maintaining analyte concentrations above the detection limit), we achieved the stated recovery rates (90–110%) in the simulated serum. Thus, sample preparation in our method is minimal and consists of a single-step sample dilution, an advantage over methods requiring complex protein extraction or precipitation.

The main limitation to the use of this method in the point-of-care format is the restricted stability of fluorescently labeled phage antibodies during storage, because they can lose their ability to bind the target during long-term storage. This is critical to a commercial point-of-care assay, which should have a shelf life of at least 6–12 months. Currently, the method has only been tested on model solutions. For use in a real point-of-care system, the device should be able to process actual samples (e.g., blood plasma). This can lead to a high background signal and to intrinsic fluorescence of plasma proteins, affecting the reliability of the analysis. Therefore, it is necessary to develop a unified sample preparation system, for example, by using a membrane filter to separate the plasma and by diluting the sample immediately before analysis, which minimizes matrix effects.

Despite the promising results in experimental models (buffer systems and simulated serum), the transition to routine clinical practice is impossible without overcoming technical obstacles related to the stability and reproducibility of phage antibodies. The main obstacles are associated not so much with sensitivity (which, as shown here, can compete with that offered by the existing methods) as with reproducibility, standardization, and automation of the process. However, the advantages of phage antibodies (low cost, stability), as combined with those of the FIA (speed and sensitivity), will help to overcome these obstacles.

## 5. Conclusions

Our results show the strong potential for using the FIA–phage antibody combination to detect HSPs. Screening studies focused on cancer marker detection with phage antibodies and the FIA open new possibilities for early cancer diagnosis and more detailed patient monitoring after tumor marker identification. The developed framework of fluorescence sensor analysis based on phage antibodies can be applied on a portable fluorimeter for measuring the content of HSPs in non-laboratory settings during mass screening.

## Data Availability

Data is contained within the article.

## Abbreviations

The following abbreviations are used in this manuscript:

CD	Circular Dichroism
FIA	Fluorescence Immunoassay
FPIA	Fluorescence Polarization Immunoassay
HSPs	Heat Shock Proteins
RecAb	Recombinant Phage Antibodies
